# Beating Tricuspid Surgery is a Viable Option with Similar Outcomes to Traditional Surgery: a Propensity Score Analysis

**DOI:** 10.21470/1678-9741-2024-0274

**Published:** 2025-10-15

**Authors:** Shoag Alahmari, Abdullah Alshehri, Juan Alfonso, Saif Ashaikmubarak, Rania Fallatah, Adam I. Adam, Khaled A. Alotaibi, Claudio Pragliola, Huda H. Ismail, Amr A. Arafat

**Affiliations:** 1 Adult Cardiac Surgery Department, Prince Sultan Cardiac Center, Riyadh, Saudi Arabia; 2 Adult Cardiac Surgery Department, Saud Albabtian Cardiac Center, Dammam, Saudi Arabia; 3 Cardiac Research Department, Prince Sultan Cardiac Center, Riyadh, Saudi Arabia; 4 Health Research Center, Ministry of Defense Health Services, Riyadh, Saudi Arabia; 5 Cardiothoracic Surgery Department, Tanta University, Tanta, Egypt

**Keywords:** Tricuspid Valve Insufficiency, Constriction, Cardiac Surgical Procedures, Heart Failure, Cardiac Arrhythmias, Intensive Care Units, Patient Readmission.

## Abstract

**Introduction:**

The advantages of beating heart tricuspid surgery without aortic cross-clamping remain underexplored, particularly in the context of concomitant procedures. This study aimed to compare the shortand long-term outcomes of tricuspid valve surgery performed with and without aortic cross-clamping.

**Methods:**

This retrospective cohort study included 1,154 patients who underwent isolated or concomitant tricuspid valve surgery between 2009 and 2021. Patients were divided into two groups, those who underwent surgery without aortic cross-clamping (beating heart, n = 170) and those with cross-clamping (arrested heart, n = 984). Propensity score matching identified 139 matched pairs.

**Results:**

The mean age was 56 years (25^th^-75^th^ percentiles: 47, 65), with 61.27% female patients and 95% undergoing concomitant procedures. In the unmatched cohort, patients who underwent beating heart surgery had higher preoperative creatinine clearance (93.53 vs. 81.33 ml/min, P = 0.036) and shorter intensive care unit stays (3 [1 - 5] vs. 3 [1 - 6] days, P = 0.037). However, after propensity score matching, there were no significant differences in postoperative heart block (P > 0.99), creatinine levels (P = 0.780), or tricuspid regurgitation grade (P = 0.082) between the two groups. Long-term outcomes, including 10-year freedom from reintervention (95% vs. 98%, log-rank P = 0.087), survival (77% vs. 82%, P = 0.964), and heart failure rehospitalization (76% vs. 77%, P = 0.444), were also comparable between the matched cohorts.

**Conclusion:**

Concomitant tricuspid surgery without aortic cross-clamping is a viable alternative to traditional arrested heart surgery, with no significant differences in shortor long-term outcomes.

## INTRODUCTION

**Table:** 

Abbreviations, Acronyms & Symbols				
AH	= Arrested heart		HF	= Heart failure
AVR	= Aortic valve replacement		IABP	= Intra-aortic balloon pump
BH	= Beating heart		ICU	= Intensive care unit
BMI	= Body mass index		MV	= Mitral valve
CABG	= Coronary artery bypass grafting		NYHA	= New York Heart Association
CHB	= Complete heart block		PAP	= Pulmonary artery pressure
COPD	= Chronic obstructive pulmonary disease		PASP	= Pulmonary artery systolic pressure
CRRT	= Continuous renal replacement therapy		RV	= Right ventricular
ECMO	= Extracorporeal membrane oxygenation		SMD	= Standardized mean difference
EDD	= End-diastolic diameter		TR	= Tricuspid regurgitation
EF	= Ejection fraction		TV	= Tricuspid valve
ESD	= End-systolic diameter		VHD	= Valvular heart disease
EuroSCORE	= European System for Cardiac Operative Risk Evaluation			

Among valvular heart diseases (VHDs), tricuspid regurgitation (TR) is considered one of the most prevalent diseases and affects 65% to 85% of VHDs patients globally^[1]^. On the other hand, tricuspid stenosis is a rare entity that affects < 1% of patients with VHD in developed countries and 3% of patients globally^[2]^. The etiology of tricuspid valve (TV) dysfunction varies between primary causes, which include rheumatic disease, congenital abnormalities, carcinoid syndrome, infective endocarditis, or iatrogenic due to intracardiac device leads, and secondary causes, which include left-sided VHD, right ventricular (RV) dysfunction, pulmonary hypertension, and atrial fibrillation^[1^,^2]^. Patients with TV disease are usually diagnosed late, remain untreated, or exhibit worse symptoms of right-sided heart failure (HF)^[3]^. The mainstay treatment for severe TV disease continues to be surgery, while medical therapy serves as a palliative measure^[1^-^3]^. However, due to late referral, patients with TV dysfunction who undergo surgery are usually at high risk and sicker and have a high mortality^[4^-^8]^.

TV surgery can be performed with either an arrested heart (AH) with cardioplegia or a beating heart (BH) without aortic cross-clamping^[5]^. BH might reduce the risk of systemic embolization associated with aortic cross-clamping, eliminate myocardial ischemia-reperfusion injury in cases of isolated TV surgery, and reduce the ischemic time in cases of concomitant TV surgery. Furthermore, the risk of heart block could be lower in patients who underwent BH TV surgery. The drawbacks of BH, on the other hand, are restricted surgical vision with blood coming from the coronary sinus, surgical manipulation being difficult due to leaflet movement, and traction of the contracting heart, which can risk tissue damage^[9^,^10]^. The debate about the potential benefits and disadvantages of beating *vs.* nonbeating TV surgery continues. Data comparing BH *vs.* AH TV surgery were mainly on isolated TV surgery, and its superiority over traditional surgery in concomitant procedures is questionable. Thus, this study aimed to characterize patients who underwent tricuspid surgery with or without an aortic cross-clamping and compare the shortand long-term outcomes.

## METHODS

### Study Design

This retrospective cohort study included 1,154 consecutive patients who underwent isolated or concomitant TV surgery between 2009 and 2021 at a single tertiary referral center. Patients were categorized into two groups depending on whether tricuspid surgery was performed (AH group [n = 984] or BH group [n = 170] without an aortic cross-clamping). The study was approved by the local ethical committee before data collection (Approval #: 1673: August 2023), and the need for informed patient consent was waived.

### Variables and Outcomes

Clinical variables in this study followed the definition of the European System for Cardiac Operative Risk Evaluation (EuroSCORE) II^[11]^. Preoperative echocardiography parameters included left ventricular systolic function, which included the left ventricular ejection fraction (EF), end-diastolic diameter (EDD), and end-systolic diameter. RV function and dilatation and pulmonary artery systolic pressure were reported^[12]^. TR was classified as five degrees: 0 = none or trace, 1 = mild, 2 = mild-moderate, 3 = moderate, and 4 = severe regurgitation.

Short-term outcomes included postoperative extracorporeal membrane oxygenation and intra-aortic balloon pump (IABP), return to the operating room for bleeding or hemodynamic compromise, dialysis or continuous renal replacement therapy, stroke, ventilation ≥ 24 hours, intensive care unit (ICU) and hospital stay, operative mortality, postoperative EF, serum creatinine, and the occurrence of complete heart block (CHB). Long-term outcomes included survival, HF rehospitalization, and TV reintervention.

### Surgical Management

The study included patients who underwent isolated or concomitant TV surgeries. TV surgery was performed without cross-clamping and cardioplegia arrest in patients who underwent isolated surgery. In patients who underwent concomitant TV surgery, the aortic clamp was removed, and rewarming started before the commencement of TV surgery. Traditional surgery was performed on cardioplegia arrest and aortic cross-clamping.

### Statistical Methods

Stata 18 (Stata Corp, College Station, Texas, United States of America) was used for analysis. Continuous data are summarized as the mean with standard deviation if normally distributed; otherwise, they are presented as the median with the 25th and 75th percentiles. The normality of the data distribution was evaluated using the Shapiro‒Wilk test. Categorical variables are presented as counts and percentages. Comparisons between groups were performed using independent *t*-tests if they were normally distributed or Mann‒Whitney U tests if they were not normally distributed. Pearson's chi-squared test was used to compare binary data. Fisher's exact test was used if the expected count was less than five. The Kaplan‒Meier method was used to report time-related events, and the log-rank test was used for comparison.

**Supplementary Fig. 1:**
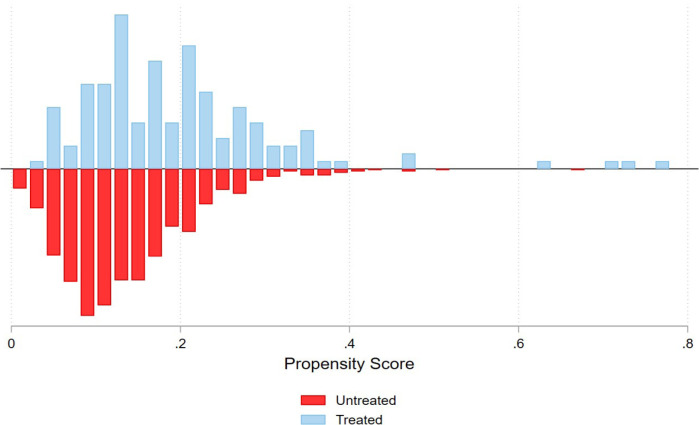
The distribution of propensity scores in the arrested heart (untreated) and beating heart (treated) groups.

**Supplementary Fig. 2:**
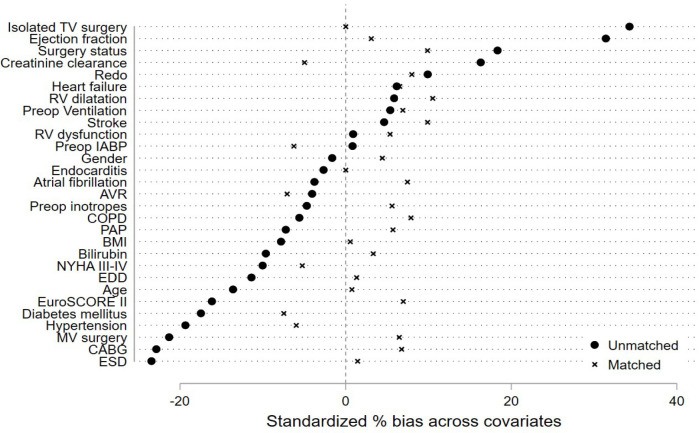
Standardized percentages of bias across covariates preand post-matching. AVR=aortic valve replacement; BMI=body mass index; CABG=coronary artery bypass grafting; COPD=chronic obstructive pulmonary disease; EDD=end-diastolic diameter; ESD=end-systolic diameter; EuroSCORE=European System for Cardiac Operative Risk Evaluation; IABP=intra-aortic balloon pump; MV=mitral valve; NYHA=New York Heart Association; PAP=pulmonary artery pressure; RV=right ventricular; TV=tricuspid valve.

Propensity score matching was used to eliminate confounders' effect on the treatment strategy choice. Nearest neighbors with 1:1 matching and a caliber of 0.02 of the standard deviations of the logits of the propensity scores were used. Patients with missing observations were excluded from propensity score matching. The distribution of propensity scores is presented in Supplementary Figure 1. An absolute percentage of standardized bias of 10% indicated satisfactory matching, and the standardized mean difference was reported (Supplementary Figure 2). Matched continuous data were compared using paired *t*-tests or Wilcoxon matched-pairs signed rank tests. Categorical data were compared using McNemar's test or the Friedman test. Time-to-event data were compared using a clustered Cox regression proportional hazard test. A two-sided *P*-value < 0.05 was considered to indicate statistical significance.

## RESULTS

### Unmatched Analysis

#### Baseline Data

The demographic data are summarized in Table 1. The median ages were 56 (25^th^-75^th^ percentiles: 47, 65) and 54 (45, 62) years for the AH and BH groups, respectively. Females comprised 61.27% of the overall cohort. Generally, similar baseline characteristics were observed between groups, except for hypertension and diabetes mellitus, which were greater in the AH group. Laboratory data revealed increased bilirubin levels and decreased creatinine clearance in the AH group. Preoperative echocardiographic measurements revealed lower EF and EDD in the AH group.

**Table 1:** Comparison of the baseline characteristics between patients who underwent arrested heart (AH) or beating heart (BH) tricuspid valve surgery in the unmatched cohort.

	Total (n = 1154)	AH (n = 984)	BH (n = 170)	*P*-value
Age (years)	56 (47, 65)	56 (47, 65)	54 (45, 62)	0.044
Female	707 (61.27%)	604 (61.38%)	103 (60.59%)	0.844
BMI (kg/m^2^)	28.23 (24.46, 32.77)	28.19 (24.5, 32.82)	28.4 1 (24.21, 32.19)	0.5835
EuroSCORE II (%) (n = 1153)	4.27 (2.33, 7.51)	4.48 (2.33,7.68)	3.64 (2.22, 6.3)	0.233
Hypertension	465 (40.29%)	410 (41.67%)	55 (32.35%)	0.022
Diabetes mellitus	456 (29.51%)	401 (40.75%)	55 (32.35%)	0.039
Endocarditis	24 (2.08%)	21 (2.13%)	3 (1.76%)	> 0.99
COPD	44 (3.81%)	39 (3.96%)	5 (2.94%)	0.520
Heart failure	136 (11.79%)	113 (11.48%)	23 (13.53%)	0.445
Stroke (n = 1153)	102 (8.85%)	85 (8.65%)	17 (10%)	0.566
Atrial fibrillation	432 (37.44%)	371 (37.70%)	61 (35.88%)	0.651
NYHA III-IV	923 (79.98%)	793 (80.59%)	130 (76.47%)	0.215
Bilirubin (mmol/L)	8 (5, 12)	8 (5, 13)	7 (5, 11)	0.0135
Creatinine clearance (ml/min) (n = 1153)	83.08 (54.23, 112.15)	81.33 (53.9, 109.61)	93.53 (55.14, 124.1)	0.0363
IABP (n = 1002)	13 (1.3%)	11 (1.28%)	2 (1.38%)	> 0.99
Mechanical ventilation (n = 1002)	9 (0.9%)	7 (0.82%)	2 (1.38%)	0.626
Inotropic support (n = 1002)	19 (1.90%)	17 (1.98%)	2 (1.38%)	> 0.99
Ejection fraction (%)	55 (40, 55)	55 (40, 55)	55 (45, 55)	0.0004
EDD (mm) (n = 1149)	52 (46, 58)	52 (46, 58)	51 (46, 56)	0.2431
ESD (mm) (n = 1148)	35 (30, 42)	36 (30, 42)	33 (30, 40)	0.0128
PASP (mmHg) (n = 1129)	50 (40, 60)	50 (40, 62.5)	50 (40, 60)	0.3541
RV dilatation	400 (34.66%)	337 (34.25%)	63 (37.06%)	0.477
RV dysfunction	234 (20.28%)	199 (20.22%)	35 (20.59%)	0.913
Degree of TR				0.086
0	6 (0.52%)	6 (0.61%)	0	
I	110 (9.53%)	93 (9.45%)	17 (10%)	
II	423 (36.66%)	371 (37.70%)	52 (30.59%)	
III	298 (25.82%)	240 (24.39%)	58 (34.12%)	
IV	317 (27.47%)	274 (27.85%)	43 (25.29%)	

The data are presented as medians (25^th^, 75^th^ percentiles) or counts (%)

BMI=body mass index; COPD=chronic obstructive pulmonary disease; EDD=end-diastolic diameter; ESD=end-systolic diameter; EuroSCORE = European System for Cardiac Operative Risk Evaluation; IABP=intra-aortic balloon pump; NYHA=New York Heart Association; PASP=pulmonary artery systolic pressure; RV=right ventricular; TR=tricuspid regurgitation

#### Operative Data

Among all cohorts, 26.78% of the patients had previous cardiac surgery; emergency surgery was indicated for 42 patients (3.64%). The majority of surgeries were tricuspid surgeries with concomitant procedures, including mitral valve (MV), aortic valve, or coronary artery bypass grafting (CABG), while isolated tricuspid surgeries were performed on only 62 subjects (5.37%). Concomitant MV surgery was the most common operation, followed by CABG. The BH group had a higher prevalence of isolated and emergency surgeries than the AH group (Table 2).

**Table 2:** Comparison of the operative data between patients who underwent arrested heart (AH) or beating heart (BH) tricuspid valve surgery in the unmatched cohort.

	Total (n = 1154)	AH (n = 984)	BH (n = 170)	*P*-value
Redo surgery	309 (26.78%)	257 (26.12%)	52 (30.59%)	0.224
Emergency surgery	42 (3.64%)	30 (3.05%)	12 (7.06%)	0.01
Isolated TV	62 (5.37%)	39 (3.96%)	23 (13.53%)	< 0.001
TV repair	1002 (86.83%)	857 (87.09%)	145 (85.29%)	0.522
Concomitant mitral valve surgery	1034 (89.6%)	892 (90.65%)	142 (83.53%)	0.005
Concomitant CABG	320 (27.73%)	287 (29.17%)	33 (19.41%)	0.009
Concomitant aortic valve surgery	247 (21.40%)	213 (21.65)	34 (20%)	0.629
Bypass time (min)	132 (103, 167)	133 (103, 167)	126.5 (104, 166)	0.3697
Cross-clamping time (n = 1140)	99.5 (75, 127)	102 (77, 129)	84 (59, 105)	< 0.001

The data are presented as medians (25^th^, 75^th^ percentiles) or counts (%)

CABG=coronary artery bypass grafting; TV=tricuspid valve

#### Short-Term Outcomes

The short-term outcomes are summarized in Table 3. Postoperative echocardiography revealed a lower EF in the AH group. The ICU stay was significantly longer in the AH group. Nonetheless, all the other early outcomes showed similar frequencies between groups. Among all patients in this study, 109 (9.45%) had operative mortality.

**Table 3:** Comparison of hospital outcomes between patients who underwent arrested heart (AH) or beating heart (BH) tricuspid valve surgery in the unmatched cohort.

	Total (n = 1154)	AH (n = 984)	BH (n = 170)	*P*-value
Open chest	64 (5.55%)	58 (5.89%)	6 (3.53%)	0.213
ECMO (n = 1143)	34 (2.97%)	30 (3.08%)	4 (2.35%)	0.807
IABP (n = 1124)	52 (4.63%)	44 (4.61%)	8 (4.73)	0.943
Re-exploration	124 (10.75%)	107 (10.87%)	17 (10%)	0.734
Dialysis/CRRT	64 (5.55%)	55 (5.59%)	9 (5.29%)	0.877
Stroke	77 (6.67%)	70 (7.11%)	7 (4.12%)	0.148
Ventilation ≥ 24 h	202 (17.5%)	174 (17.68%)	28 (16.47%)	0.701
ICU stay	3 (1, 6)	3 (1, 6)	3 (1, 5)	0.037
Hospital stay (n = 1153)	12 (8, 21)	12 (8, 21)	10.5(7, 20)	0.144
Operative mortality	109 (9.45%)	91 (9.25%)	18 (10.59%)	0.581
Ejection fraction (%) (n = 1152)	50 (40, 55)	50 (40, 55)	55 (46, 55)	0.002
Creatinine (mmol/l)	70 (56-92)	70 (56-92)	68 (56-81)	0.351

The data are presented as medians (25^th^, 75^th^ percentiles) or counts (%)

CRRT=continuous renal replacement therapy; ECMO=extracorporeal membrane oxygenation; IABP=intra-aortic balloon pump; ICU=intensive care unit

#### Long-Term Outcomes

**Fig. 1:**
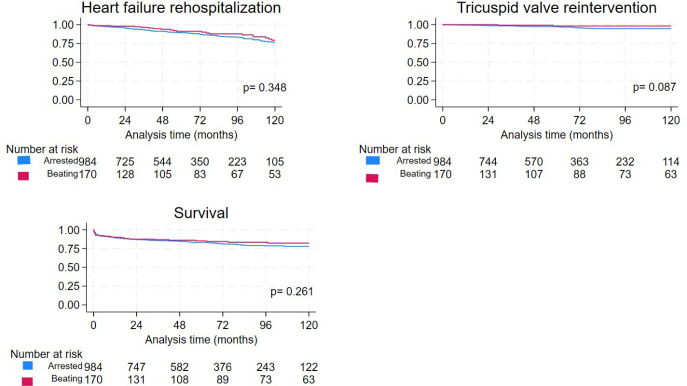
Freedom from heart failure rehospitalization and tricuspid valve reintervention and survival in beating patients vs. arrested heart tricuspid valve surgery patients in the unmatched cohort.

In the AH group, 118 patients experienced HF rehospitalization, while in the BH group, 23 patients experienced HF rehospitalization. Freedom from HF rehospitalization in the AH group was 89% and 76% *vs.* 91% and 77% in the BH group at five and 10 years, respectively (log-rank *P* = 0.348) (Figure 1).

Of the total AH group, 31 subjects underwent TV reintervention *vs.* two in the BH group. Among all reinterventions, 23 were surgical reinterventions *vs.* 10 via the transcatheter approach. Freedom from TV reintervention in AH patients was 97% at five years and 95% at 10 years, while in the BH group, it was 98% at both five and 10 years (*P* = 0.087) (Figure 1).

Survival analysis for the AH group *vs.* the BH group showed similar results; 175 deaths occurred in the AH group, while 26 deaths occurred in the BH group. Survival was 82% and 77% in the AH group, while in the BH group, it was 85% and 82% at five years and 10 years, respectively (*P* = 0.261) (Figure 1).

#### Matched Analysis

Propensity score matching identified 139 matched pairs. A comparison of the baseline and operative data of the matched cohort is presented in Table 4.

**Table 4:** Comparison of the baseline characteristics between patients who underwent arrested heart (AH) or beating heart (BH) tricuspid valve surgery in the matched cohort.

	AH (n = 139)	BH (n = 139)	SMD
Age (years)	54 (46, 64)	55 (45, 63)	-0.007
Female	80 (57.55%)	83 (59.71%)	-0.04
BMI (kg/m^2^)	28.06 (23.97, 32.05)	28.82 (24.38, 32.21)	-0.005
EuroSCORE II (%)	2.88 (1.71, 5.27)	3.52 (2.02, 5.93)	-0.09
Hypertension	48 (34.53%)	44 (31.65%)	0.06
Diabetes mellitus	48 (34.53%)	43 (30.94%)	0.07
Endocarditis	0	0	-
COPD	1 (0.72%)	3 (2.16%)	-0.12
Heart failure	18 (12.95%)	21 (15.11%)	-0.06
Stroke	10 (7.19%)	14 (10.07%)	-0.10
Atrial fibrillation	39 (28.06%)	44 (31.65%)	-0.07
NYHA III-IV	115 (82.73%)	112 (80.58%)	0.05
Bilirubin (mmol/L)	7 (6, 12)	8 (5, 12)	-0.04
Creatinine clearance (ml/min)	100.22 (75.76, 127.33)	102.12 (75.8, 128.92)	0.06
IABP	2 (1.44%)	1 (0.72%)	0.07
Ventilation	1 (0.72%)	2 (1.44%)	-0.07
Inotropic support	1 (0.72%)	2 (1.44%)	-0.07
Ejection fraction (%)	55 (45, 55)	55 (45, 55)	-0.03
EDD (mm)	52 (46, 57)	52 (47, 57)	-0.01
ESD (mm)	35 (29, 40)	34 (30, 40)	-0.01
PASP (mmHg)	50 (40, 60)	50 (40, 60)	-0.06
RV dilatation	31 (22.3%)	38 (27.34%)	-0.12
RV dysfunction	16 (11.51%)	19 (13.67%)	-0.06
Degree of TR			-0.10
0	1 (0.72%)	0	
I	19 (13.67%)	16 (11.51%)	
II	55 (39.57%)	48 (34.53%)	
III	24 (17.27%)	36 (25.90%)	
IV	40 (28.78%)	39 (28.06%)	
Redo surgery	26 (18.71%)	31 (22.30%)	-0.09
Emergency surgery	4 (2.88%)	7 (5.04%)	-0.11
Isolated TV	5 (3.60%)	5 (3.60%)	0
TV repair	139 (100%)	139 (100%)	0
Concomitant mitral valve	128 (92.09%)	131 (94.24%)	-0.09
Concomitant CABG	25 (17.99%)	29 (20.86%)	-0.07
Concomitant aortic valve	33 (23.74%)	29 (20.86%)	0.07
Bypass time (min)	123 (93, 155)	123 (106, 165)	-0.05
Cross-clamping time (min)	97 (71, 127)	84 (60, 105)	0.35

The data are presented as medians (25^th^, 75^th^ percentiles) or counts (%)

BMI=body mass index; CABG=coronary artery bypass grafting; COPD=chronic obstructive pulmonary disease; EDD=end-diastolic diameter; ESD=end-systolic diameter; EuroSCORE=European System for Cardiac Operative Risk Evaluation; IABP=intra-aortic balloon pump; NYHA=New York Heart Association; PASP=pulmonary artery systolic pressure; RV=right ventricular; SMD=standardized mean difference; TR=tricuspid regurgitation; TV=tricuspid valve

Among the matched pairs, the BH group had a longer hospital stay and more patients who needed IABP postoperatively, but neither difference reached statistical significance. CHB occurred in five patients in each group (*P* > 0.99). There were no differences in hospital outcomes between the groups (Table 5). When we compared preoperative TR measurements to postoperative measurements, there was a significant improvement in the degree of TR in both groups (*P* < 0.001).

**Table 5:** Comparison of hospital outcomes between patients who underwent arrested heart (AH) or beating heart (BH) tricuspid valve surgery in the matched cohort.

	AH (n = 139)	BH (n = 139)	*P*-value
Open chest	6 (4.32%)	5 (3.60%)	> 0.99
ECMO	3 (2.17%)	3 (2.16%)	> 0.99
IABP	2 (1.47%)	7 (5.07%)	0.180
Re-exploration	9 (6.47%)	11 (7.91%)	0.804
Dialysis/CRRT	6 (4.32%)	5 (3.60%)	> 0.99
Stroke	6 (4.32%)	6 (4.32%)	> 0.99
Ventilation ≥ 24 h	21 (15.11%)	18 (12.95%)	0.720
ICU stay	2 (1, 4)	2 (1, 4)	0.942
Hospital stay	8 (6, 17)	10 (7, 17)	0.265
Operative mortality	12 (8.63%)	11 (7.91%)	> 0.99
Ejection fraction (%)	55 (45, 55)	55 (45, 55)	0.739
Post TR			0.082
0	73 (52.52%)	53 (38.13%)	
I	46 (33.09%)	70 (50.36%)	
II	15 (10.79%)	11 (7.91%)	
III	4 (2.88%)	4 (2.88%)	
IV	1 (0.72%)	1 (0.72%)	
Creatinine (mmol/l)	70 (56-92)	68 (55-80)	0.780

The data are presented as medians (25^th^, 75^th^ percentiles) or counts (%)

CRRT=continuous renal replacement therapy; ECMO=extracorporeal membrane oxygenation; IABP=intra-aortic balloon pump; ICU=intensive care unit; TR=tricuspid regurgitation

**Fig. 2:**
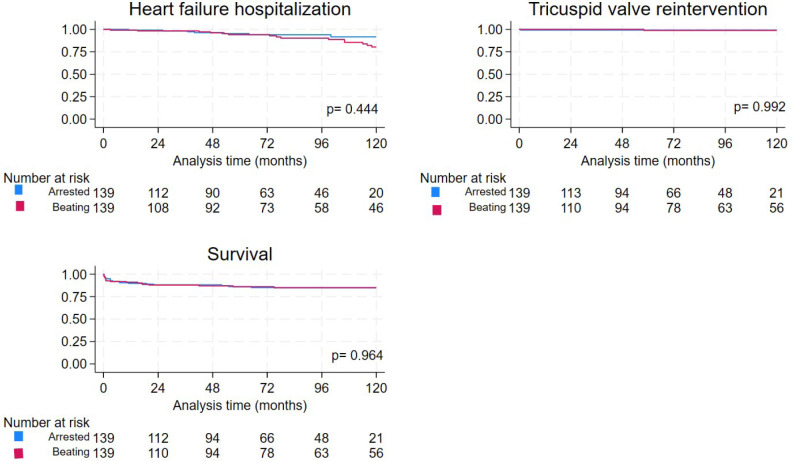
Freedom from heart failure rehospitalization and tricuspid valve reintervention and survival in beating patients vs. arrested heart tricuspid valve surgery patients in the matched cohort.

Long-term outcome analysis revealed that both the AH and BH groups had similar rates of freedom from HF rehospitalization, TV reintervention, and long-term survival (Figure 2).

## DISCUSSION

Despite the increasing interest in TV, several aspects of tricuspid surgery remain to be investigated^[2^,^13^,^14]^. Although the current practice for severe TR is more often concomitant surgery, especially left-heart surgery, the benefits of beating tricuspid surgery have been evaluated in isolated procedures^[15^-^17]^. In a study from the Society of Thoracic Surgeons Adult Cardiac Surgery Database, Chen et al. demonstrated the beneficial effects of beating isolated TV surgery in reducing the need for pacemakers, renal failure, and blood transfusion^[17]^. However, the value of concomitant TV surgery without aortic cross-clamping has not been investigated. This study compared the shortand long-term outcomes of beating *vs.* arrested TV surgery using propensity score matching analysis. The study demonstrated that AH patients were older and had a greater prevalence of diabetes and hypertension and lower creatinine clearance. After matching, there was no difference between groups in postoperative outcomes, including operative mortality, creatinine level, TR grade, and heart block. Furthermore, the long-term outcomes did not differ significantly between the two techniques.

In a study by Russo et al., where 129 pairs of propensity-matched analyses of isolated TV surgeries were performed at 13 international sites, the BH group showed a greater incidence of new atrial fibrillation postoperatively than the AH group^[5]^. However, longer cardiopulmonary bypass and ischemic times were reported to be risk factors for postoperative atrial fibrillation^[18]^. This finding may indicate that atrial fibrillation could be related to ischemic time rather than cardiopulmonary bypass time. On the other hand, the pacemaker implantation rate was not different, which was similar to our study. Similarly to the findings of Chen et al., a study by Russo showed a greater renal failure rate in arrested hearts^[5^,^17]^. Flagiello et al. reported similar shortand long-term outcomes between BH and AH isolated TV surgery, although BH patients were at greater risk, with higher EuroSCORE II and redo surgery^[10]^. Furthermore, the study reported higher but nonsignificant heart block in patients who underwent AH surgery (17% *vs.* 6%), which is inconsistent with our findings. Pfannmüller et al. reported significantly older patients and females in the BH group, with a greater prevalence of redo surgeries and atrial fibrillation[9]. This finding contrasts with our data since AH patients were older and had more comorbidities. Pfannmüller et al. reported no difference between BH and AH regarding the degree of TR, which is consistent with our findings^[9]^. This indicates a similar efficiency of both techniques in repairing the TV. Beating tricuspid surgery might be beneficial in a subgroup of patients who undergo redo surgery. Several studies have demonstrated the potential benefits of BH TV surgery in redo patients^[19^,^20]^, a finding that was not confirmed in our study since the outcomes did not differ between BH and AH in patients who underwent redo surgery.

We did not observe differences in the long-term outcomes between the two techniques. This finding is in contrast to that of the Baraki study, which reported greater TV reintervention and lower survival in patients who underwent BH-isolated TV surgery^[21]^. Similarly, Russo et al. reported a higher rate of the composite endpoint of cardiac death and reoperation in patients who underwent BH surgery^[5]^. Furthermore, they reported a significantly lower survival rate and greater cardiac event rate with TV replacement than with repair, which could be attributed to the longer cardiopulmonary bypass time required in patients who underwent TV replacement^[5]^. The propensity score matching in our study excluded all replacement patients, and the comparison was made on TV repair only. The five-year survival rates in the Flagiello study were 80% in the BH group and 85% in the AH group, and the five-year survival rates at 10 years were 73.4% and 42.5% in the BH and AH groups, respectively^[10]^. In our study, survival rates were 82% and 77% in the AH group and 85% and 82% in the BH group at five years and 10 years, respectively.

The study's findings demonstrated no added benefits of concomitant TV surgery performed without cross-clamping compared with traditional surgery. These data may indicate that the results of studies on isolated tricuspid surgery should not be generalized to tricuspid surgery concomitant with other procedures. Beating tricuspid surgery could be beneficial in mitigating the risk of isolated high-risk surgery^[22]^.

### Limitations

Our study acknowledges the limitations inherent in its retrospective and observational design but provides consecutive data over 12 years of clinical practice. Although this was a single-center study, we did not apply any protocol regarding surgical approach or indications, both of which were based on different surgeons' decisions. In our attempt to eliminate any differences among participants, our sample size decreased substantially. Furthermore, several unmeasured confounders might have had an effect and were not included in the analysis.

## CONCLUSION

Our findings suggest that although concomitant tricuspid surgery without an aortic cross-clamping might be a viable option, it has no additional beneficial effects compared to traditional surgery on an arrested heart. We recommend further investigations through randomized clinical trials to validate these results.

## Data Availability

The authors declare that the data will be available upon reasonable request to the authors after approval of the Institutional Review Board on data sharing.

## Artificial Intelligence Usage

The authors declare that no artificial intelligence tool was used in the preparation of this article.
